# Histiocytic sarcoma progressing from follicular lymphoma and mimicking acquired hemophagocytic lymphohistiocytosis

**DOI:** 10.1007/s00277-020-04190-4

**Published:** 2020-08-17

**Authors:** Christoph Schünemann, Gudrun Göhring, Yvonne Lisa Behrens, Hans-Heinrich Kreipe, Arnold Ganser, Felicitas Thol

**Affiliations:** 1 Department of Hematology, Hemostasis, Oncology, and Stem Cell Transplantation, Hannover Medical School, Carl-Neuberg-Str.1, 30625 Hannover, Germany; 2 Department of Human Genetics, Hannover Medical School, Carl-Neuberg-Str.1, 30625 Hannover, Germany; 3 Institute of Pathology, Hannover Medical School, Carl-Neuberg-Str.1, 30625 Hannover, Germany

Dear Editor,

Histiocytic sarcoma (HS) is a rare and aggressive non-Langerhans cell histiocytosis with a poor prognosis [1]. In a subset of cases, HS is clonally related to concurrent hematological malignancies, especially lymphomas [2]. Acquired hemophagocytic lymphohistiocytosis (HLH) is a systemic reactive process that includes macrophage activation with hemophagocytosis in the bone marrow [3].

A 57-year-old woman presented with a progressive hypopharyngeal tumor. B symptoms were absent, and laboratory results were unremarkable. Histologic examination showed a blastic infiltration with monocytoid/histiocytic morphology with CD68, CD163, CD14, and lysozyme expression (CD1a, CD3, CD20, CD34, CD56, CD117, MPO and S100 negative, immunohistochemistry (IHC)) (Fig. 1a). Staging revealed mediastinal, mesenterial, and retroperitoneal lymphadenopathy as well as an incidental follicular lymphoma (FL) grade 1 in the bone marrow with otherwise unaffected hematopoiesis. Histiocyte infiltration of the bone marrow was excluded. Fluorescence in situ hybridization (FISH) revealed the presence of translocation t(14;18) within the cervical HS as well as the FL of the bone marrow (Fig. 1b). Immunoglobulin heavy chain (IgH) rearrangement was present in the FL but absent in the cervical HS (PCR amplification). We diagnosed HS probably arising from follicular lymphoma.

**Fig. 1:**
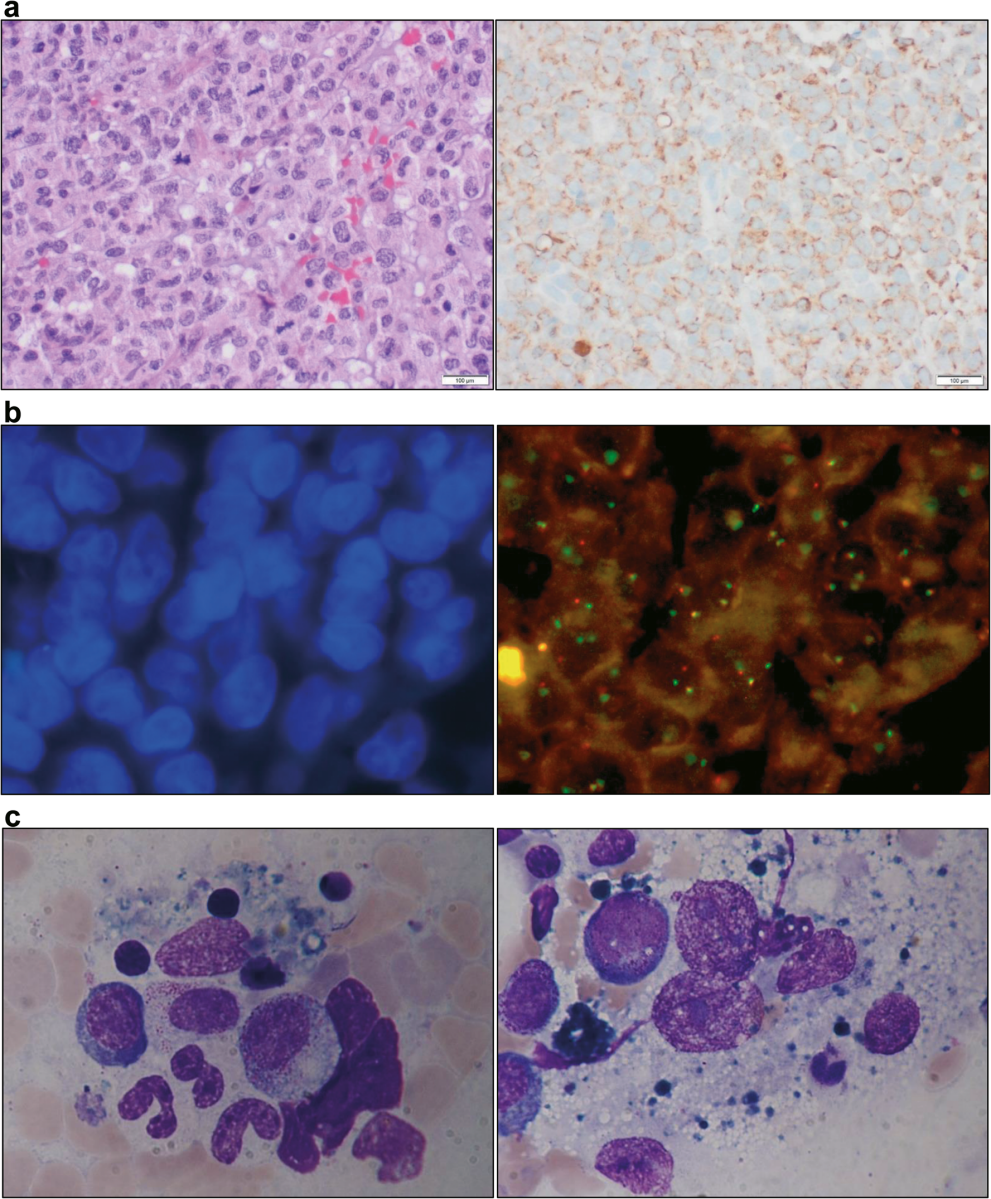
Histopathological and cytological studies during course of disease. **a** Histopathology images of a biopsy of a cervical mass with blastic infiltrate and histiocytic sarcoma (HS) immunophenotype. Left: Hematoxylin and eosin (H&E). Right: CD68 immunohistochemical staining (original magnification × 400, bars represent 100 μm). **b** FISH analysis of histiocytic sarcoma identified translocation t(14;18). Left: Nuclear staining with DAPI. Right: BCL2 FISH break apart probe (ZytoLight SPEC BCL2, Zytovision, Bremerhaven, Germany). Orange/green fusion signal indicated non-rearranged BCL2 gene. Orange and green separate signals confirmed rearranged BCL2 gene (original magnification × 1000). **c** Day 54 bone marrow aspirate showed prominent wide and polygonal histiocytes with large nuclei and 1–3 prominent nucleoli. The cytoplasm was slightly basophilic and foamy with prominent hemophagocytosis. Pappenheim stain (original magnification × 1000)

We initiated a therapy with R-CHOEP (rituximab, cyclophosphamide, doxorubicin, vincristine, prednisone with etoposide) and documented a stable disease after two cycles of therapy.

Subsequently, therapy continuation was delayed due to persisting fever. Extensive diagnostics and empiric antimicrobial therapies failed. The patient’s general condition markedly worsened with onset of a sepsis-like syndrome with a high fever, tachycardia, and hypotension as well as an unexplained progressive pancytopenia. Another bone marrow aspirate showed slightly increased cellularity, normal hematopoiesis, and extensive hemophagocytosis (Fig. 1c).

Because of the clinical presentation and positive diagnostic criteria for HLH according to the HLH-2004 protocol [4] (5 out of 8 criteria, including rising ferritin of 7370 μg/L), we suspected malignancy-associated HLH (mal-HLH). We initiated corticosteroids promptly (dexamethasone 10 mg/m^2^) on day 54 after chemotherapy initiation but observed progressive pancytopenia and hemodynamic instability over the subsequent days. According to current recommendations [5] for mal-HLH, we continued to treat the underlying malignancy with the third course of R-CHOEP. Despite these measures, the patient deteriorated and died shortly afterwards in a distributive shock.

Further work-up of the histopathological examination of the second bone marrow sample revealed t(14;18) positive HS infiltration and prominent hemophagocytosis within malignant histiocytes (FISH, IHC). The clinical presentation that mimicked secondary HLH was based on refractory HS with bone marrow infiltration and neoplastic hemophagocytosis in the proper sense.

Clinically relevant cytopenia due to hemophagocytosis in HS has been rarely described [6–8]. The combination of hemophagocytosis with pancytopenia and inflammatory response syndrome has not been linked to fulminant HS bone marrow infiltration. This finding is relevant for clinical management of HS. HS may be considered differential diagnosis for inflammatory syndromes with hemophagocytosis such as HLH, especially when refractory to standard therapy.

## Data Availability

Not applicable.
